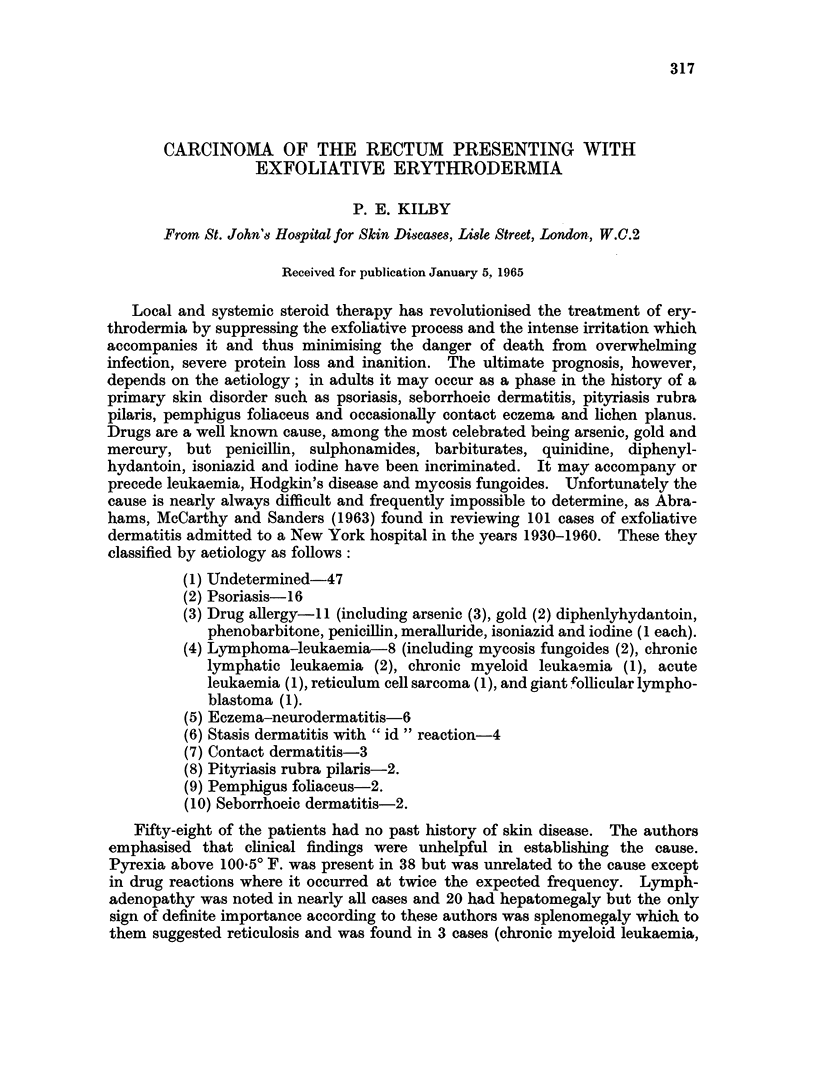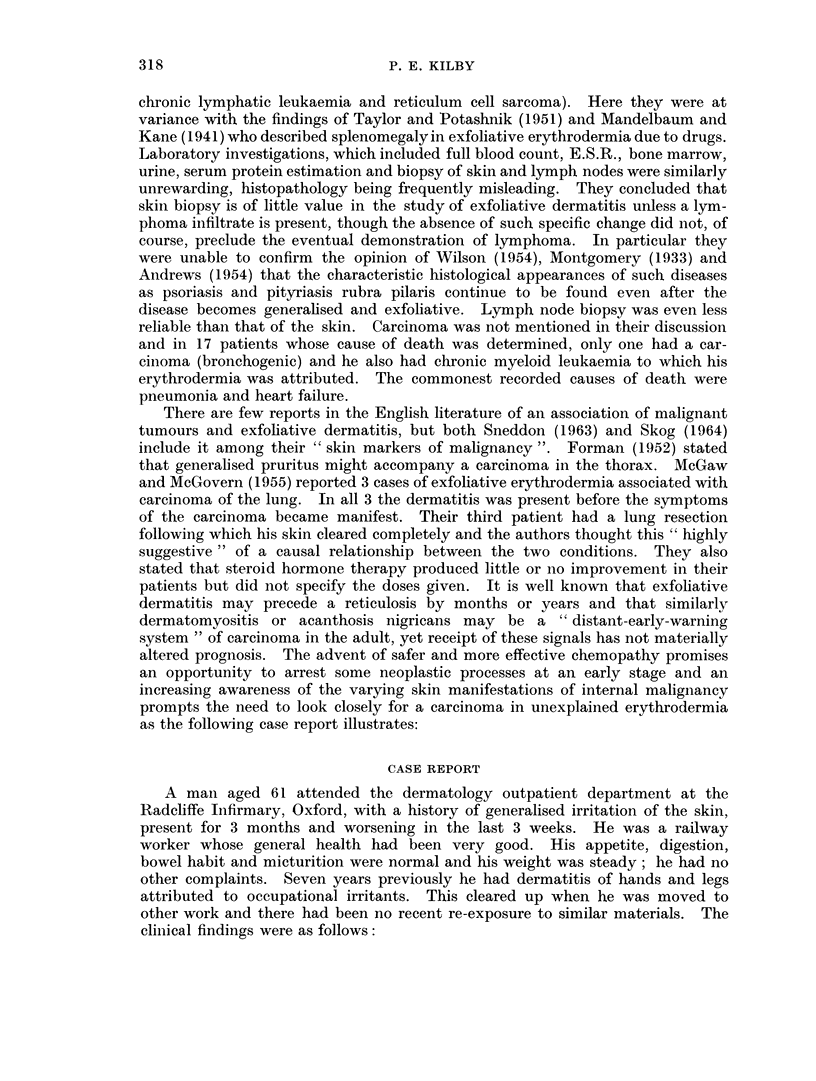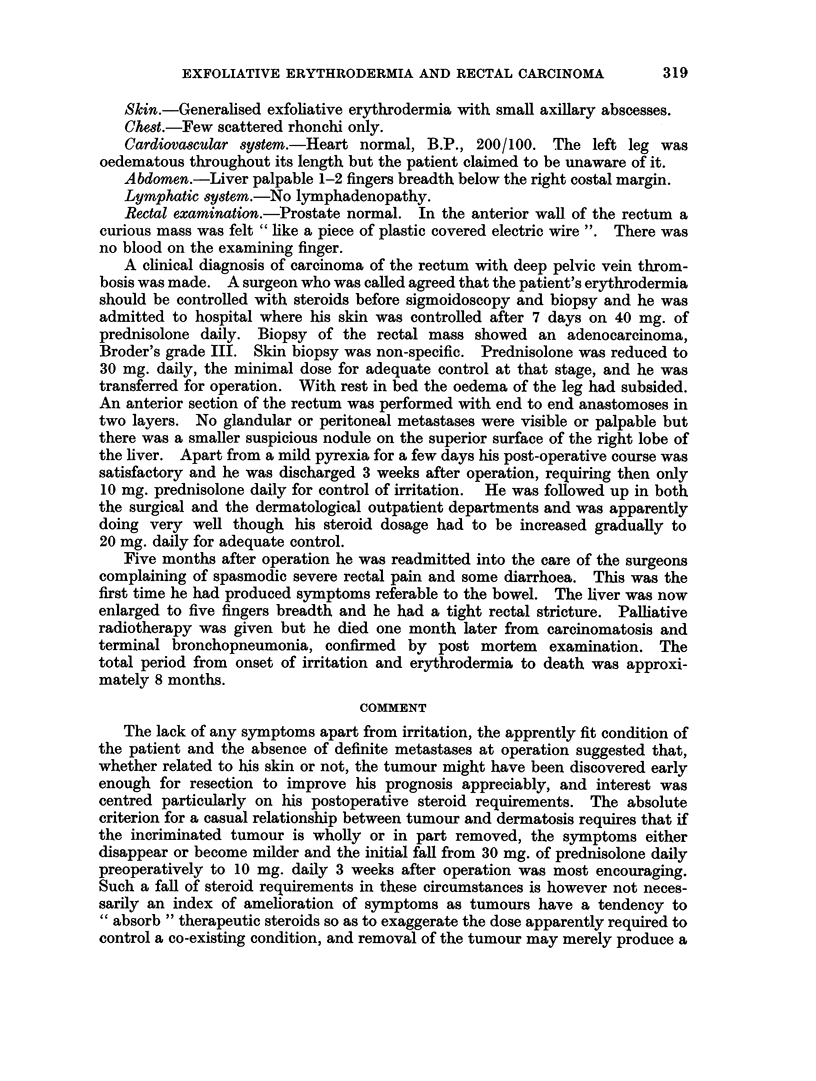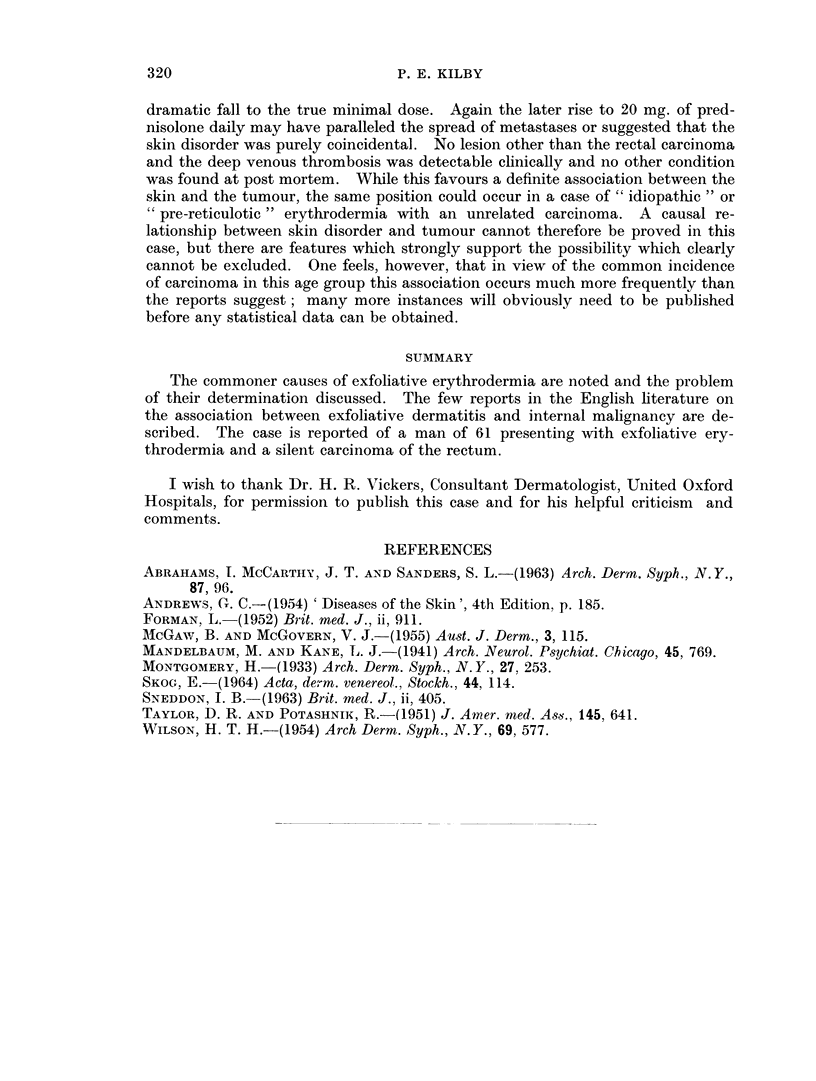# Carcinoma of the Rectum Presenting with Exfoliative Erythrodermia

**DOI:** 10.1038/bjc.1965.36

**Published:** 1965-06

**Authors:** P. E. Kilby


### CARCINOMA OF THE RECTUM PRESENTING WITH EXFOLIATIVE ERYTHRODERMIA

P. E. KILBY

*From St. John's Hospital for Skin Diseases, Lisle Street, London, W.C.2*

Received for publication January 5, 1965

Local and systemic steroid therapy has revolutionised the treatment of erythrodermia by suppressing the exfoliative process and the intense irritation which accompanies it and thus minimising the danger of death from overwhelming infection, severe protein loss and inanition. The ultimate prognosis, however, depends on the aetiology ; in adults it may occur as a phase in the history of a primary skin disorder such as psoriasis, seborrhoeic dermatitis, pityriasis rubra pilaris, pemphigus foliaceus and occasionally contact eczema and lichen planus. Drugs are a well known cause, among the most celebrated being arsenic, gold and mercury, but penicillin, sulphonamides, barbiturates, quinidine, diphenylhydantoin, isoniazid and iodine have been incriminated. It may accompany or precede leukaemia, Hodgkin's disease and mycosis fungoides. Unfortunately the cause is nearly always difficult and frequently impossible to determine, as Abrahams, McCarthy and Sanders (1963) found in reviewing 101 cases of exfoliative dermatitis admitted to a New York hospital in the years 1930–1960. These they classified by aetiology as follows :

(1) Undetermined—47
(2) Psoriasis—16
(3) Drug allergy—11 (including arsenic (3), gold (2) diphenlyhydantoin, phenobarbitone, penicillin, meralluride, isoniazid and iodine (1 each).
(4) Lymphoma–leukaemia—8 (including mycosis fungoides (2), chronic lymphatic leukaemia (2), chronic myeloid leukaemia (1), acute leukaemia (1), reticulum cell sarcoma (1), and giant follicular lymphoblastoma (1).
(5) Eczema–neurodermatitis—6
(6) Stasis dermatitis with " id " reaction—4
(7) Contact dermatitis—3
(8) Pityriasis rubra pilaris—2.
(9) Pemphigus foliaceus—2.
(10) Seborrhoeic dermatitis—2.

Fifty-eight of the patients had no past history of skin disease. The authors emphasised that clinical findings were unhelpful in establishing the cause. Pyrexia above 100·5° F. was present in 38 but was unrelated to the cause except in drug reactions where it occurred at twice the expected frequency. Lymphadenopathy was noted in nearly all cases and 20 had hepatomegaly but the only sign of definite importance according to these authors was splenomegaly which to them suggested reticulosis and was found in 3 cases (chronic myeloid leukaemia, chronic lymphatic leukaemia and reticulum cell sarcoma). Here they were at variance with the findings of Taylor and Potashnik (1951) and Mandelbaum and Kane (1941) who described splenomegaly in exfoliative erythrodermia due to drugs. Laboratory investigations, which included full blood count, E.S.R., bone marrow, urine, serum protein estimation and biopsy of skin and lymph nodes were similarly unrewarding, histopathology being frequently misleading. They concluded that skin biopsy is of little value in the study of exfoliative dermatitis unless a lymphoma infiltrate is present, though the absence of such specific change did not, of course, preclude the eventual demonstration of lymphoma. In particular they were unable to confirm the opinion of Wilson (1954), Montgomery (1933) and Andrews (1954) that the characteristic histological appearances of such diseases as psoriasis and pityriasis rubra pilaris continue to be found even after the disease becomes generalised and exfoliative. Lymph node biopsy was even less reliable than that of the skin. Carcinoma was not mentioned in their discussion and in 17 patients whose cause of death was determined, only one had a carcinoma (bronchogenic) and he also had chronic myeloid leukaemia to which his erythrodermia was attributed. The commonest recorded causes of death were pneumonia and heart failure.

There are few reports in the English literature of an association of malignant tumours and exfoliative dermatitis, but both Sneddon (1963) and Skog (1964) include it among their " skin markers of malignancy ". Forman (1952) stated that generalised pruritus might accompany a carcinoma in the thorax. McGaw and McGovern (1955) reported 3 cases of exfoliative erythrodermia associated with carcinoma of the lung. In all 3 the dermatitis was present before the symptoms of the carcinoma became manifest. Their third patient had a lung resection following which his skin cleared completely and the authors thought this " highly suggestive " of a causal relationship between the two conditions. They also stated that steroid hormone therapy produced little or no improvement in their patients but did not specify the doses given. It is well known that exfoliative dermatitis may precede a reticulosis by months or years and that similarly dermatomyositis or acanthosis nigricans may be a " distant-early-warning system " of carcinoma in the adult, yet receipt of these signals has not materially altered prognosis. The advent of safer and more effective chemopathy promises an opportunity to arrest some neoplastic processes at an early stage and an increasing awareness of the varying skin manifestations of internal malignancy prompts the need to look closely for a carcinoma in unexplained erythrodermia as the following case report illustrates:

### CASE REPORT

A man aged 61 attended the dermatology outpatient department at the Radcliffe Infirmary, Oxford, with a history of generalised irritation of the skin, present for 3 months and worsening in the last 3 weeks. He was a railway worker whose general health had been very good. His appetite, digestion, bowel habit and micturition were normal and his weight was steady ; he had no other complaints. Seven years previously he had dermatitis of hands and legs attributed to occupational irritants. This cleared up when he was moved to other work and there had been no recent re-exposure to similar materials. The clinical findings were as follows :

*Skin.*—Generalised exfoliative erythrodermia with small axillary abscesses.

*Chest.*—Few scattered rhonchi only.

*Cardiovascular system.*—Heart normal, B.P., 200/100. The left leg was oedematous throughout its length but the patient claimed to be unaware of it.

*Abdomen.*—Liver palpable 1–2 fingers breadth below the right costal margin.

*Lymphatic system.*—No lymphadenopathy.

*Rectal examination.*—Prostate normal. In the anterior wall of the rectum a curious mass was felt " like a piece of plastic covered electric wire ". There was no blood on the examining finger.

A clinical diagnosis of carcinoma of the rectum with deep pelvic vein thrombosis was made. A surgeon who was called agreed that the patient's erythrodermia should be controlled with steroids before sigmoidoscopy and biopsy and he was admitted to hospital where his skin was controlled after 7 days on 40 mg. of prednisolone daily. Biopsy of the rectal mass showed an adenocarcinoma, Broder's grade III. Skin biopsy was non-specific. Prednisolone was reduced to 30 mg. daily, the minimal dose for adequate control at that stage, and he was transferred for operation. With rest in bed the oedema of the leg had subsided. An anterior section of the rectum was performed with end to end anastomoses in two layers. No glandular or peritoneal metastases were visible or palpable but there was a smaller suspicious nodule on the superior surface of the right lobe of the liver. Apart from a mild pyrexia for a few days his post-operative course was satisfactory and he was discharged 3 weeks after operation, requiring then only 10 mg. prednisolone daily for control of irritation. He was followed up in both the surgical and the dermatological outpatient departments and was apparently doing very well though his steroid dosage had to be increased gradually to 20 mg. daily for adequate control.

Five months after operation he was readmitted into the care of the surgeons complaining of spasmodic severe rectal pain and some diarrhoea. This was the first time he had produced symptoms referable to the bowel. The liver was now enlarged to five fingers breadth and he had a tight rectal stricture. Palliative radiotherapy was given but he died one month later from carcinomatosis and terminal bronchopneumonia, confirmed by post mortem examination. The total period from onset of irritation and erythrodermia to death was approximately 8 months.

### COMMENT

The lack of any symptoms apart from irritation, the apprently fit condition of the patient and the absence of definite metastases at operation suggested that, whether related to his skin or not, the tumour might have been discovered early enough for resection to improve his prognosis appreciably, and interest was centred particularly on his postoperative steroid requirements. The absolute criterion for a casual relationship between tumour and dermatosis requires that if the incriminated tumour is wholly or in part removed, the symptoms either disappear or become milder and the initial fall from 30 mg. of prednisolone daily preoperatively to 10 mg. daily 3 weeks after operation was most encouraging. Such a fall of steroid requirements in these circumstances is however not necessarily an index of amelioration of symptoms as tumours have a tendency to " absorb " therapeutic steroids so as to exaggerate the dose apparently required to control a co-existing condition, and removal of the tumour may merely produce a dramatic fall to the true minimal dose. Again the later rise to 20 mg. of prednisolone daily may have paralleled the spread of metastases or suggested that the skin disorder was purely coincidental. No lesion other than the rectal carcinoma and the deep venous thrombosis was detectable clinically and no other condition was found at post mortem. While this favours a definite association between the skin and the tumour, the same position could occur in a case of " idiopathic " or " pre-reticulotic " erythrodermia with an unrelated carcinoma. A causal relationship between skin disorder and tumour cannot therefore be proved in this case, but there are features which strongly support the possibility which clearly cannot be excluded. One feels, however, that in view of the common incidence of carcinoma in this age group this association occurs much more frequently than the reports suggest ; many more instances will obviously need to be published before any statistical data can be obtained.

SUMMARY

The commoner causes of exfoliative erythrodermia are noted and the problem of their determination discussed. The few reports in the English literature on the association between exfoliative dermatitis and internal malignancy are described. The case is reported of a man of 61 presenting with exfoliative erythrodermia and a silent carcinoma of the rectum.

I wish to thank Dr. H. R. Vickers, Consultant Dermatologist, United Oxford Hospitals, for permission to publish this case and for his helpful criticism and comments.

### REFERENCES

Abrahams, I. McCarthy, J. T. and Sanders, S. L.—(1963) *Arch. Derm. Syph., N.Y.*, **87**, 96.
Andrews, G. C.—(1954) ' Diseases of the Skin ', 4th Edition, p. 185.
Forman, L.—(1952) *Brit. med. J.*, ii, 911.
McGaw, B. and McGovern, V. J.—(1955) *Aust. J. Derm.*, **3**, 115.
Mandelbaum, M. and Kane, L. J.—(1941) *Arch. Neurol. Psychiat. Chicago*, **45**, 769.
Montgomery, H.—(1933) *Arch. Derm. Syph., N.Y.*, **27**, 253.
Skog, E.—(1964) *Acta, derm. venereol., Stockh.*, **44**, 114.
Sneddon, I. B.—(1963) *Brit. med. J.*, ii, 405.
Taylor, D. R. and Potashnik, R.—(1951) *J. Amer. med. Ass.*, **145**, 641.
Wilson, H. T. H.—(1954) *Arch Derm. Syph., N.Y.*, **69**, 577.